# Congenital Obstructive Hydrocephalus With Status Post-endoscopic Third Ventriculostomy Bilateral Subdural Hygroma and Pneumocephalus: A Case Report

**DOI:** 10.7759/cureus.65982

**Published:** 2024-08-02

**Authors:** Sakshi Desai, H. V. Sharath

**Affiliations:** 1 Department of Pediatric Physiotherapy, Ravi Nair Physiotherapy College, Datta Meghe Institute of Higher Education and Research, Wardha, IND

**Keywords:** pediatrics, infant, endoscopic third ventriculostomy, pneumocephalus, hydrocephalus

## Abstract

Pediatric neurosurgery faces a major difficulty in the treatment of hydrocephalus, a condition marked by an abnormal build-up of cerebrospinal fluid (CSF) in the brain. Its prevalence varies between 0.5 and 0.8 per 1,000 live births worldwide, with different etiologies, including congenital abnormalities and acquired diseases. With benefits including a lower risk of infection and avoiding issues due to the shunt, endoscopic third ventriculostomy (ETV) has become a beneficial surgical technique in certain instances. Bypassing clogged ventricular channels, ETV creates a new channel for CSF drainage. Despite its effectiveness, a thorough examination of underlying disease and anatomical variables is necessary for positive outcomes in patient selection. To give patients, the best possible care, this article attempts to summarize the prevalence of hydrocephalus and the part that ETV plays in managing it. It also emphasizes the significance of customized surgical techniques. It is critical to comprehend the incidence of hydrocephalus and available treatment choices to enhance the infant's quality of life and long-term outcomes.

## Introduction

The name hydrocephalus was not coined until the works of Celsus, who lived between 25 BC and 50 AD. Hippocrates is credited with the earliest documented accounts of "water" surrounding the brains of macrocephalic children [1-3]. When cerebrospinal fluid (CSF) is not properly absorbed into the systemic circulation from its place of generation inside the ventricular system, it can cause hydrocephalus, an active distension of the brain's ventricular system [4]. The obstructive kind of posterior fossa tumors include cerebellum-pontine angle tumors, posterior third ventricle tumors, hydrocephalus related to congenital aqueduct stenosis, and other tumors. The most common types of hydrocephalus are obstructive ones, which include encephalocele, craniosynostosis, syringomyelia with or without Chiari malformation, hydrocephalus after cerebellar infarct, and intraventricular hematoma. Additionally, indicative of obstructive hydrocephalus includes multiloculate hydrocephalus, slit ventricle syndrome, and myelomeningocele with hydrocephalus [5].

A significant rise in head circumference is the initial clinical sign of hydrocephalus in neonates and infants. An increase in the cranial cavity's capacity makes up for the CSF rise, provided that the fontanelle and cranial sutures are not fused. Additional distinctive symptoms include enlarged and protruding anterior fontanelles, low eye and ear placement, separation of the cranial sutures, and dilated veins, which are especially noticeable on a thin and shining scalp. The "sunset eye sign," often referred to as the "setting sun phenomenon," is characterized by downcast eyes, and Macewen's sign, which is a cracked pot sound, is another classic symptom of hydrocephalus [6].

In recent times, endoscopic methods have been utilized to treat several neurological diseases [7]. It is well acknowledged that obstructive hydrocephalus of different etiologies can be treated with endoscopic third ventriculostomy (ETV) [8]. Reducing intracranial pressure (ICP) enhances neurological function, which is the aim of ETV [9]. ETV is a minimally invasive neurosurgery technique that involves making a tiny hole in the third ventricle's floor so that the CSF may pass through the blockage and flow freely [10]. Improvement of symptoms without requiring further shunts is referred to as successful ETV. Furthermore, the operation is now safer, more effective, and requires less recuperation time due to advancements in endoscopic technology. On the other hand, ventricular posterior shunt (VPS) is a surgical technique in which a catheter is inserted to drain CSF into the peritoneal cavity, where it is reabsorbed [11,12]. This case report talks about a seven-month-old infant diagnosed with congenital obstructive hydrocephalus with bilateral subdural hygroma and pneumocephalus. The article aims to provide insights into the successful management of congenital obstructive hydrocephalus and improving the infant’s quality of life.

## Case presentation

A 2.2 kg male baby was born to a gravida 2, parity 2, live 2, abortion 0 female with no history of consanguinity, attempted abortion, diabetes, hypertension, thyroid issues, emotional trauma, or convulsions. The mother had regular antenatal care visits. The baby was born through normal vaginal delivery. The baby cried immediately after birth. The baby has no history of neonatal intensive care unit stay. The baby's activity was normal after the birth. At the age of six months, the child had recurrent episodes of vomiting, and the parents noticed swelling around the head, for which the parents consulted a general physician. The physician prescribed medications and advised for MRI brain. The head size of the baby rapidly increased. On February 10, 2024, the child was brought to our hospital, where further examination was conducted. The laboratory findings are described in Table 1.

**Table 1:** Laboratory findings RBC: red blood cells; HB: hemoglobin; MCV: mean corpuscular volume; WBC: white blood cells; HCT: hematocrit; MCHC: mean corpuscular hemoglobin concentration; RDW: red cell distribution width; APTT: activated partial thromboplastin time; mm³: cubic millimeter; gm/dL: grams per deciliter

Laboratory Findings	Values	Normal Values
RBC	5.8 million cells per microliter (cells/mcL)	4.7-6.1 million cells per microliter (cells/mcL)
HB	11.7% gm/dL	11.3-14.3 gm/dL
MCV	63.8 femtolitres	70-85 femtolitres
Total RBC count	5.5 million red blood cells per microliter of blood	4.7-6.1 million red blood cells per microliter of blood
Total WBC count	19400/mm^3^	5000-19000/mm^3^
HCT	37%	31-41%
MCHC	31.7 gm/dL	23-31 gm/dL
RDW	16.6%	15.5-20%
Monocytes	04%	2-8%
Lymphocytes	45%	20-40%
Eosinophils	01 cells per microliter of blood	0-450 cells per microliter of blood
Basophils	0 cells per microliter of blood	0-200 cells per microliter of blood
Coagulation profile revealed APTT-control	29.5 seconds	25-35 seconds
APTT-patient	29.8 seconds	25-35 seconds
Prothrombin time-control	11.9 seconds	12.5-15.2 seconds
Prothrombin time-patient	12 seconds	12.5-15.2 seconds

Investigatory findings

The MRI brain reveals dilatation of the lateral ventricles on both sides, indicating ventricular enlargement. This finding suggests the presence of hydrocephalus, which may be due to impaired CSF flow or increased CSF production. There is evidence of diffuse changes in the brain parenchyma, indicating widespread abnormalities in the brain tissue. These changes could be due to various causes, including hypoxic-ischemic injury, infections, or metabolic disorders. The presence of a subdural hygroma is noted, which is a collection of CSF within the subdural space. This can result from trauma, surgery, or spontaneous leakage and may contribute to increased ICP or neurological symptoms.

**Figure 1:**
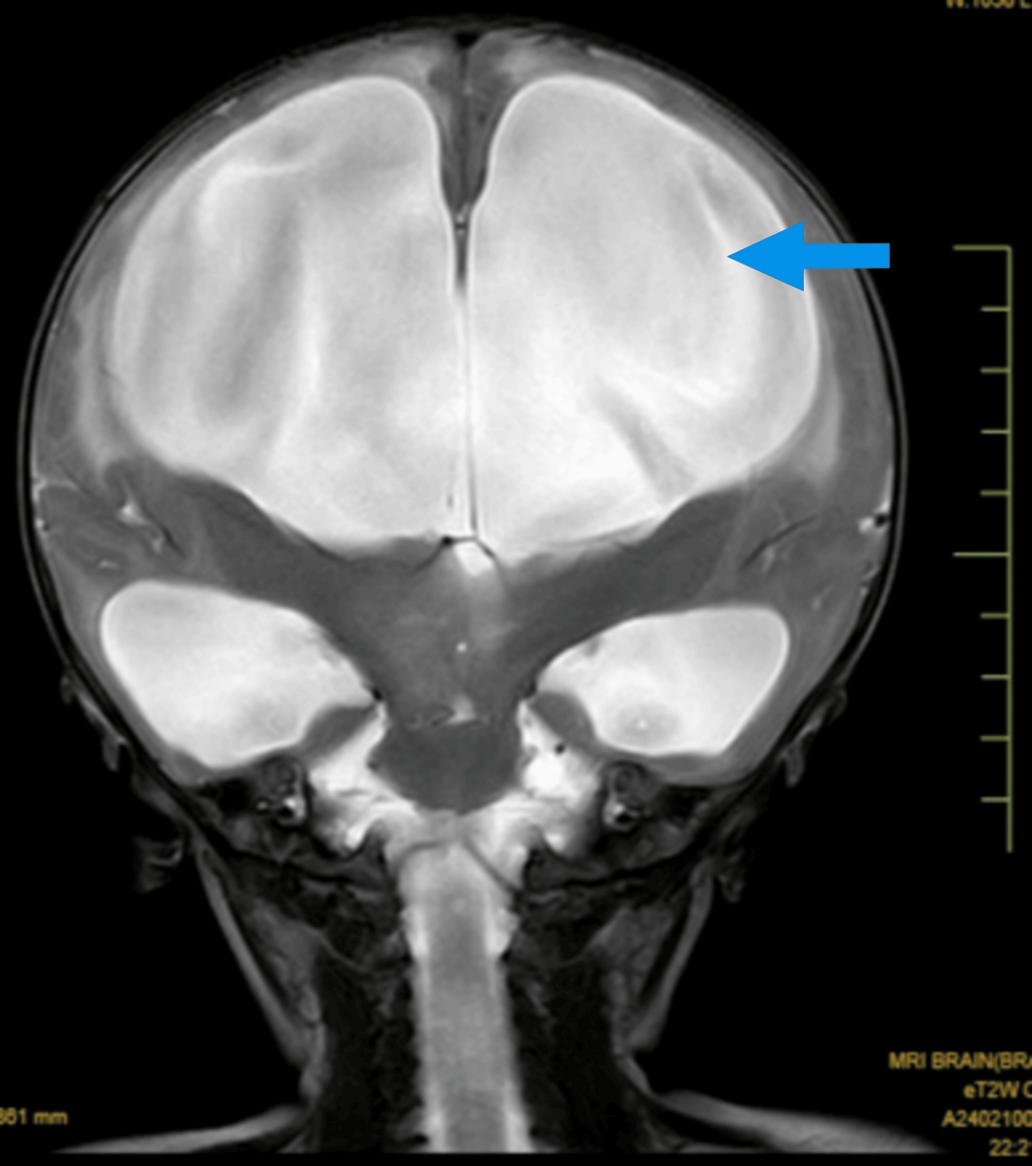
MRI brain showing hydrocephalus with subdural hygroma and pneumocephalus The arrow indicates dilatation of bilateral ventricles and diffuse involvement of the brain parenchyma along with the presence of subdural hygroma and pneumocephalus.

These MRI findings suggest significant neurological involvement and require prompt clinical correlation and intervention. The presence of dilated ventricles, diffuse brain parenchyma changes, subdural hygroma, and pneumocephalus indicates a complex clinical scenario that may necessitate multidisciplinary management, including neurology, neurosurgery, and critical care. The MRI film is given in Figure 1.

Surgical procedure

A durotomy and corticotomy were done, and scope with sheath was introduced. The Fogarty catheter (Edwards Lifesciences, Irvine, USA) was used to perform the aforementioned fenestration. Hemostasis and free flow of CSF were confirmed. The scope was taken out. The wound was closed in layers. Dressing was done. After that, the child was diagnosed with congenital obstructive hydrocephalus with status post-endoscopic third ventriculostomy (S/P ETV) bilateral subdural hygroma. On February 28, 2024, keens point subdural-peritoneal low-pressure shunt placement was done. Under strict aseptic precautions, a right keens point curvilinear incision was made, and the dura was exposed after nibbling the bone with tension rangers. Abdominal incision made dissected layer by layer to reach peritoneum.

Subcutaneous tunneling was done. Chhabra low-pressure shunt (Chhabra Medical Devices, New Delhi, India) was prepared and inserted through the tunnel. A durotomy was done. The shunt catheter was fixed and connected to the reservoir end. The free flow of CSF was confirmed. The abdominal end was placed in a peritoneal cavity under direct vision. Hemostasis was confirmed in both wounds. Wounds were closed in layers. The skin was closed with Monocryl (Ethicon, Inc., Somerville, USA). Dressing was done. The child was kept in the pediatric intensive care unit for observation.

On examination

Post-surgery, the first examination was done after taking informed consent and informed assent. The child was examined in a supine lying position. A general physical examination revealed a pulse rate of 138bpm, respiratory rate of 38b/m, SPO2 of 100%, and blood pressure of 102/58mmhg; the child was afebrile. Anthropometrics findings revealed length is 70cm; head circumference is 49cm and chest circumference is 34cm. On observation, the body type is ectomorphic. The typical setting sun appearance is seen in the infant. The anterior fontanelle is wide, tense, and elevated. The visual tracking of the objects is absent. The chest is bilaterally symmetrical, and the baby exhibited an abdominothoracic pattern of breathing. Cyanosis, clubbing, and lymphadenopathy were absent. On auscultation, air entry was bilaterally equal. On assessment of gross motor developmental milestones, neck control still needs to be achieved.

## Discussion

This case report focuses on a seven-month-old male child with congenital obstructive hydrocephalus who underwent ETV and subsequently developed bilateral subdural hygroma with pneumocephalus. The postoperative outcomes, both in terms of complications and improvements in clinical features, provide valuable insights into the efficacy and challenges associated with ETV in young children. Hydrocephalus in infants often presents with characteristic clinical features such as macrocephaly, frontal bossing, bulging anterior fontanelle, and increased muscle tone. Parents typically report symptoms like poor feeding, irritability, and vomiting, with the most critical indicator being the progressively increasing head circumference. These symptoms were observed in our patient, necessitating the surgical intervention of ETV to alleviate the condition.

ETV is generally preferred over VPS due to its lower complication rates and fewer long-term impacts on the quality of life. The literature indicates a high frequency (20-80%) of significant postoperative complications associated with VPS, which often result in reduced quality of life for patients. In contrast, ETV offers a less invasive alternative with a comparatively lower risk of complications [13]. However, the age of the patient has a significant impact on the success rate of ETV. According to Kadrian et al., the effectiveness of ETV in infants under one month old is particularly low, with only 41% of those aged one to six months maintaining a functioning ETV after five years. This rate improves significantly with age, with 58% of patients aged six to 24 months and over 70% of those older than 24 months experiencing long-term success. These findings are corroborated by other studies, emphasizing the critical role of patient age in determining the outcome of ETV. In this case, the seven-month-old patient demonstrated notable postoperative improvements [14-16].

The seven-month-old patient in this instance showed significant surgical improvements. The baby's capacity to visually track things and begin gross motor milestones like rolling improved significantly after the ETV. Furthermore, there was a noticeable improvement in eating patterns, a reduction in irritability, and notable advancements in the gross motor activities of the upper and lower extremities, such as the beginning of object gripping [17,18]. These encouraging results imply that the ETV was successful in reducing the hydrocephalus symptoms and advancing the child's developmental milestones, even in spite of the problems of bilateral subdural hygroma and pneumocephalus. Pneumocephalus and subdural hygroma developing as postoperative sequelae are serious concerns. Changes in ICP dynamics after ETV may give rise to these disorders. In this instance, nonetheless, the child's recoveries and enhanced motor abilities as well as general well-being suggest that these issues were successfully managed [19,20].

In summary, even if there is a chance of postoperative complications including subdural hygroma and pneumocephalus, this case illustrates the possible advantages of ETV in treating congenital obstructive hydrocephalus in infants. The patient's improvements highlight the significance of comprehensive postoperative monitoring and care to guarantee the best possible results. Given the substantial influence of patient age on the efficacy of endoscopic therapy (ETT), this example further emphasizes the need for comprehensive parental counseling and well-informed decision-making in the treatment of hydrocephalus.

## Conclusions

Congenital obstructive hydrocephalus with bilateral subdural hygroma and pneumocephalus is a complicated condition. Appropriate assessment, evaluation, diagnosis, and surgical method selection can provide positive results and enhance the quality of life for an infant.
